# The Totally Extraperitoneal Method versus Lichtenstein's Technique for Inguinal Hernia Repair: A Systematic Review with Meta-Analyses and Trial Sequential Analyses of Randomized Clinical Trials

**DOI:** 10.1371/journal.pone.0052599

**Published:** 2013-01-11

**Authors:** G. G. Koning, J. Wetterslev, C. J. H. M. van Laarhoven, F. Keus

**Affiliations:** 1 Department of Surgery, Radboud University Nijmegen Medical Centre, Nijmegen, The Netherlands; 2 The Copenhagen Trial Unit (CTU), Centre of Clinical Intervention Research, University of Copenhagen, Rigshospitalet, Copenhagen, Denmark; Sapienza University of Rome, Italy

## Abstract

**Background:**

Lichtenstein's technique is considered the reference technique for inguinal hernia repair. Recent trials suggest that the totally extraperitoneal (TEP) technique may lead to reduced proportions of chronic pain. A systematic review evaluating the benefits and harms of the TEP compared with Lichtenstein's technique is needed.

**Methodology/Principal Findings:**

The review was performed according to the ‘Cochrane Handbook for Systematic Reviews’. Searches were conducted until January 2012. Patients with primary uni- or bilateral inguinal hernias were included. Only trials randomising patients to TEP and Lichtenstein were included. Bias evaluation and trial sequential analysis (TSA) were performed. The error matrix was constructed to minimise the risk of systematic and random errors. Thirteen trials randomized 5404 patients. There was no significant effect of the TEP compared with the Lichtenstein on the number of patients with chronic pain in a random-effects model risk ratio (RR 0.80; 95% confidence interval (CI) 0.61 to 1.04; *p* = 0.09). There was also no significant effect on number of patients with recurrences in a random-effects model (RR 1.41; 95% CI 0.72 to 2.78; *p* = 0.32) and the TEP technique may or may not be associated with less severe adverse events (random-effects model RR 0.91; 95% CI 0.73 to 1.12; *p* = 0.37). TSA showed that the required information size was far from being reached for patient important outcomes.

**Conclusions/Significance:**

TEP versus Lichtenstein for inguinal hernia repair has been evaluated by 13 trials with high risk of bias. The review with meta-analyses, TSA and error matrix approach shows *no conclusive* evidence of a difference between TEP and Lichtenstein on the primary outcomes chronic pain, recurrences, and severe adverse events.

## Introduction

Inguinal hernia repair is one of the most frequently performed procedures in surgery and many different techniques have been suggested. Techniques vary essentially by: using a mesh or not, the position of the mesh (onlay, inlay or sublay), the approach of the hernia (anterior or posterior), and the technique being open or endoscopic. It has been shown that the use of a mesh is associated with a reduced number of patients with recurrence [1].

Both a systematic review and a meta-analysis without a systematic review have been published [1], [2]. In these, combinations of different techniques are compared in one intervention group versus combinations of other techniques as a control group. However, one specific technique for inguinal hernia repair cannot be claimed to be superior based on the comparisons of heterogeneous intervention groups [3].

Guidelines in many West European countries consider the Lichtenstein technique as the reference standard [4]. Recent reports suggest that a preperitoneal mesh, by the endoscopic totally extraperitoneal (TEP) method, results in a reduction of chronic pain and a quicker recovery [2]. Conceptually, the TEP rather than the transabdominal preperitoneal (TAPP) approach seems a logic choice as it avoids entering the abdominal cavity.

A systematic review of randomized trials comparing only the TEP technique versus only the Lichtenstein technique is needed. Available evidence needs to be evaluated in the perspective of the three dimensions of possible risks of errors: the systematic error (bias), the random error (‘the play of chance’), and the design error (the outcome measure chosen).

The objective was to conduct a systematic review of the benefits and harms of the TEP technique compared with the Lichtenstein technique for inguinal hernia repair.

## Methods

This review was conducted according to the prior published protocol following the recommendations of the ‘Cochrane Handbook for Systematic Reviews’ [3] and reported according to the PRISMA statement (at: www.prisma-statement.org). The protocol [5] of this review is available online at http://www.ctu.dk.

### Criteria for considering studies for this review

#### Studies

Only randomized trials were considered for inclusion irrespective language, blinding, publication status, or sample size. It was intended not to include quasi-randomized trials regarding assessment of benefits, but it was intended to include regarding assessment of harms [3].

#### Patients

Only adult patients were considered. Patients with primary uni- or bilateral inguinal hernias were included, but patients with hernia repair for recurrent hernias were excluded since proportions of patients with chronic pain may differ.

#### Interventions

Trials using the TEP technique by endoscopy and any type of mesh for inguinal hernia repair were included. Trials using the transabdominal preperitoneal (TAPP) technique were excluded. Trials using both the TEP and TAPP technique were included only if the vast majority of more than 80% of interventions were performed with the TEP technique.

The Lichtenstein technique using any type of mesh was considered the control intervention; trials using any other open technique were excluded.

#### Outcomes

The outcome measures were graded according to the patients' perspective (GRADE working group 2004) [6].

Primary outcomes were all-cause mortality, chronic pain defined as persisting pain for longer than three months, recurrences, and severe adverse events (SAE).

The composite outcome measure of SAE outlined in the protocol in advance, was constructed summarizing all severe complications including chronic pain, deep wound infections, vascular injuries, visceral injuries, and recurrences [5]. It was recognized that the number of complications may have been summarized rather than the number of patients with one or more SAE. Therefore, double counts may have occurred. Since severe complications in elective hernia repair are rather rare, it is expected that double counts will be limited to less than 5%.

Secondary outcomes were conversions, time until return to usual activity, length of hospital stay, and duration of operation [5]. Other secondary outcomes were reported according to availability of data.

#### Search strategy

Searches included MeSH descriptors (“Clinical Trials”, “Randomized Controlled Trials”, “TEP”, “TEPP”, “totally, extraperitoneal”, “Lichtenstein”, “Liechtenstein”, “laparoscopic”, “Laparoscopy”, “preperitoneal”, “endoscopic”, “inguinal hernia”, “Hernia, Inguinal”) and were performed in CENTRAL on The Cochrane Library (Issue 1 2012), The National Library of Medicine (MEDLINE/PubMed) (1966–January 2012), and The Intelligent Gateway to Biomedical & Pharmacological Information (EMBASE) (1980–January 2012) for randomized trials (Appendix S1). Additional relevant trials were looked for by checking the reference lists of identified reviews and randomized trials.

#### Data collection and analysis

Two authors independently identified trials for inclusion and extracted the following data: year and language of publication, country in which the trial was conducted, duration of the trial, single- or multicenter design, and in- and exclusion criteria. Further, baseline imbalance and early stopping of trials were registered. All trial authors were requested for additional information lacking in their reports. Any differences in opinion were resolved through discussion.

#### Assessment of bias risk

The risk of bias of the trials was assessed by two authors independently, without masking of trial names, following the instructions given in the *Cochrane Handbook for Systematic Reviews of Interventions* [3]. According to empirical evidence [7]–[10], risk of bias components were scored as low, unclear, or high. The following risk of bias components were extracted from each trial: generation of the allocation sequence, allocation concealment, blinding (of participants, personnel, and outcome assessors), incomplete outcome data, selective outcome reporting, and other bias risks such as academic bias and source of funding bias.

Trials were classified as trials with low risk of bias only if all risk of bias components were scored as low. Otherwise, if one or more of the bias components were scored unclear or with high risk of bias, the trial was considered to have a high risk of bias.

#### Error matrix approach

Data on the outcomes of all trials were assessed for the risk of bias (measured by the level of evidence), the risk of random error measured by standard error (SE), and the design error measured by grading the outcomes [11]. Data were presented in a three-dimensional Manhattan error matrix which may facilitate the overview of available evidence at a glance and may identify possible lacunae.

#### Statistical analysis

Meta-analyses were performed according to the *Cochrane Handbook for Systematic Reviews of Interventions* [3] using Review Manager version 5.1 [12].

For a dichotomous variable, the risk ratio (RR) with the 95% confidence interval (CI) was calculated if there were two or more trials for an outcome. For events occurring less than 5% the odds ratios (OR) with their 95% CI were calculated. The proportion of patients with the outcome in each group and the *p*-value for the comparison between the groups was reported. For continuous variables, the mean difference (MD) or the standardized mean difference (SMD) with 95% CI were calculated. For both dichotomous and continuous outcomes a *p*-value of less than 0.05 was considered statistically significant.

A random-effects model [13] and a fixed-effect model [14] were used for meta-analysis in the presence of two or more trials included under the outcomes. In case of discrepancy between the two models, both results were reported. Considering the anticipated abundant clinical heterogeneity the random-effects model was emphasized except if one or two trials dominated the available evidence. Heterogeneity was explored by Cochran's test. Significance was set at *p*-value 0.10, and the quantity of heterogeneity was measured by I^2^ [3], [15]. The analyses were performed on an intention-to-treat basis whenever possible. Otherwise, the ‘available-case analysis’ was adopted [3]. No data for the post-randomization drop-outs for any of the continuous outcomes was imputed [16].

#### Sensitivity analyses

In sensitivity analyses the standard deviation was imputed from *p-*values according to the instructions given in the *Cochrane Handbook for Systematic Reviews of Intervention* and the median was used for the meta-analysis when the mean was not available [3]. If it was not possible to calculate the standard deviation from the *p-*value or the confidence interval, the standard deviation was imputed as the highest standard deviation noted for that group under that outcome.

#### Subgroup analyses

It was intended to perform the following subgroup analyses:

Trials with low risk of bias (all bias components scored as low risk) compared to trials with high risk of bias (one or more of the bias components scored as unclear or high risk). Trials were divided in two groups based on the time of publication. Results of an initial first group were compared to the results of the second (last) group to evaluate whether results have improved over time. Only subgroup analyses showing statistical significant test of interaction (*p*<0.05) provided evidence that the intervention effect may depend on the subgroup.

#### Bias exploration

It was planned to use a funnel plot to explore small trial bias [17], [18] and to use asymmetry in funnel plot of trial size against treatment effect to assess this bias.

#### Trial sequential analysis

Cumulative meta-analyses may increase type-I errors due to sparse data and repeated significance testing when updated with new trials [19], [20]. To minimise the risk of type-I errors, trial sequential analysis (TSA) was used. TSA combines an estimation of the required information size for a meta-analysis (meta-analysis sample size) with an adjusted threshold for statistical significance of the meta-analysis [19]–[21]. The latter, called trial sequential monitoring boundaries (TSMB), reduce the risk of type-I errors. In TSA the addition of a new trial in a cumulative meta-analysis is regarded as an interim meta-analysis and helps to clarify whether additional trials are needed or not. The idea in TSA is that when the cumulative *z*-curve crosses the TSMB, a sufficient level of evidence has been reached and no further trials may be needed. If the *z*-curve doesn't cross one of the boundaries for benefit, harm or futility and the required information size has not been reached, there is insufficient evidence to reach a conclusion [19], [20], [22], [23]. Information size was calculated as diversity-adjusted required information size [24] based on an a priori anticipated [5] relative risk reduction of 20% and by the relative risk reduction of the intervention effect suggested in a meta-analysis of the included trials. TSA was performed on all primary outcomes and on all secondary outcomes showing statistically significant differences between the two interventions. The required information size was calculated according to an overall type-I error of 5% and a power of 80% [24]. The analyses were performed using the TSA program and manual, developed by The Copenhagen Trial Unit (CTU, Center for Clinical Intervention Research, Denmark). The TSA software and manual are available at: www.ctu.dk/tsa.

## Results

**Figure 1:**
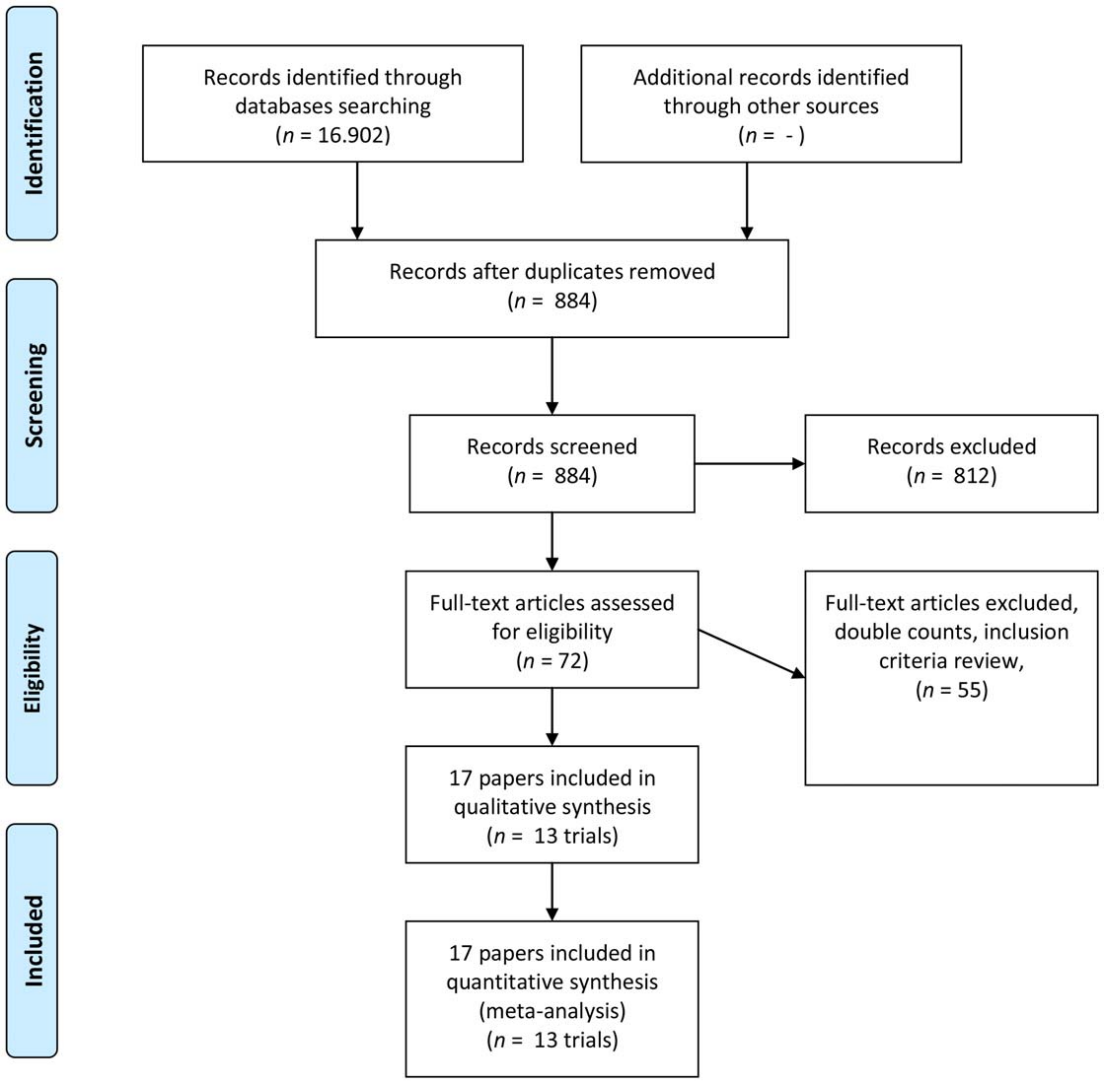
Flow diagram summarizing the search process and results of each phase of the systematic review.

Altogether the search resulted in 16.902 hits. In each step of selection, the publication was included in case of any doubt. A total of 884 hits remained after manual screening of the titles. All abstracts were reviewed independently by two authors. Double publications of trial results were considered as one trial. Based on titles and abstracts 812 publications could be excluded. A total of 72 publications remained for full text evaluation from which 55 were excluded based on the protocol criteria. Finally, seventeen publications describing 13 randomized trials were included (Figure 1). Additional data of each trial was requested by contacting the investigators. None of the included trials used quasi-randomized design.

### Patient characteristics and trial designs

All 13 trials used similar inclusion criteria. The specifications of the exclusion criteria varied. From one of the trials information was only available as a poster [25]. Arguments for imbalances in baseline characteristics regarding age, gender, BMI, or ASA classification were not found (Table 1). One study [26], [27] consisted of three trials; only the trial comparing TEP versus Lichtenstein was selected. All other trials used a two-arm parallel-group design [25], [26], [28]–[41].

**Table 1:** Baseline Characteristics of randomized TEP-and Lichtenstein of all included trials.

Author	Included patients (n)	Included patients (n)		Multi/Single Center	Age (yr)	Gender		ASA Classification	(I/II/III/IV)
*TEP*	*Lichtenstein*	*TEP*		*Lichtenstein*	*TEP*	*Lichtenstein*		*TEP*	*Lichtenstein*
Andersson 2003 [28]	81	87	S	50.0 (SD9)	49.0 (SD9)	M	M	C	C
Colak 2003 [29]	67	67	S	49.4 (R21–78)	51.6 (R16–77)	M57/F10 pts	M62/F5 pts	C	C
Eklund 2006 [30]–[33]	665	706	M	53.0 (SD10)	52.0 (SD10)	M	M	584/66/5/0 pts	633/57/5/0 pts
Gokalp 2003 [34]	61	62	S	47.0 (R18–59)	45.0 (R18–60)	M	M	72/28/0/0%	64.5/35.5/0/0%
Heikkinen 1998 [26]	23	22	S	44.0 (R21–65)	46.0 (R22–58)	M	M	68/32/0/0%	70/30/0/0%
Hildebrandt 2003 [35]	72	66	S	54.5(SD13.6)	60.0 (SD12.7)	M51/F4 pts	M57/F9 pts	Mean ASA 1.82	Mean ASA 1.49
Merello 1997 [25]	60	60	S	Median 53	Median 51	U	U	C	C
Moreno 1999 [36]	50	50	S	55.0 (R21–80)	60.0(R24–73)	M47/F3 pts	M44/F6 pts	U	U
Neumayer 2004 [37]	1077	1087	M	58.6 (SD12.8)	58.4 (SD12.7)	M	M	34.7/46.8/18.5/0%	33.6/47.7/18.7/0%
Lal 2003 [38]	25	25	S	36.7(SD12.1)	37.8 (SD12.4)	M	M	C	C
Langeveld 2010 [39]	336	324	M	Median 55	Median 56	M99%	M98%	Mean 1	Mean 1
Lau 2006 [40]	100	100	S	55 (SD15.5)	56 (SD13.1)	M	M	C	C
Wright 1996 [41]	67	64	M	63.0 (R46–71)	68.0 (R51–77)	M56/F4 pts	M59/F1 pts	37/22/1/0 pts	29/28/3/0 pts

### Surgical interventions

In all trials the TEP hernia repair was performed as published by Voeller [42]. The Lichtenstein technique was performed as described by Amid [43], [44]. One trial had a mixed group of TEP and TAPP procedures [37]. However, this trial was included since 90% of the patients were operated with the TEP technique according to personal communication with the author. Open procedures in all trials were Lichtenstein repairs.

### Risk of bias

**Figure 2:**
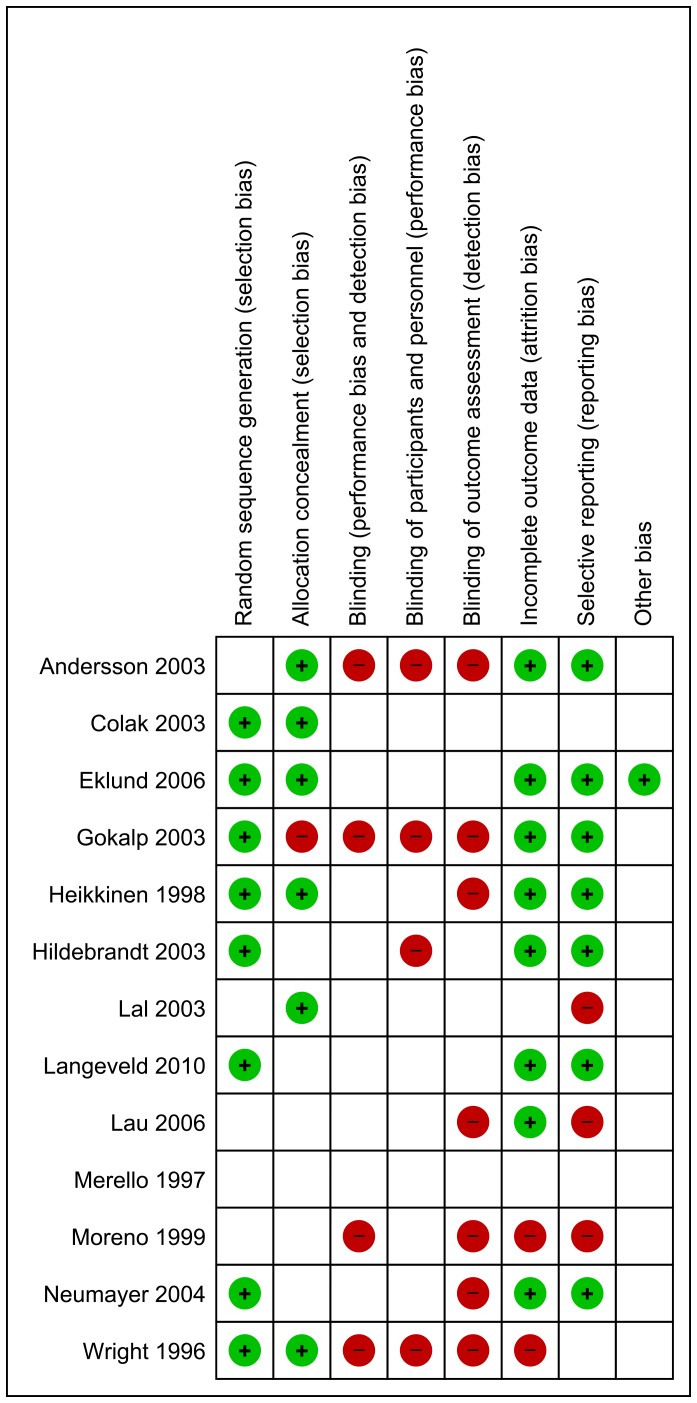
risk of bias summary of all included trials, the eight criteria on the X-axis. Name of first author and year of trial on Y-axis. + = adequate. − = inadequate. Blanc = unclear.

The risk of bias of the included trials was assessed (Figure 2) [3], [12]. Many bias risk components were unclear. None of the trials used any form of blinding, especially no blinding of outcome assessment. In all trials three or more out of eight bias components were scored as unclear or high risk of bias. Therefore, all trials were classified as high risk of bias trials.

### Error matrix approach

In clinical research there are 3 dimensions that have widely been recognized to be important.

The included trials were assessed for risks of errors: the risk of bias measured by the level of evidence, the risk of random error measured by standard error, and the design error measured by grading the outcome measures according to GRADE [6], [11].

**Figure 3:**
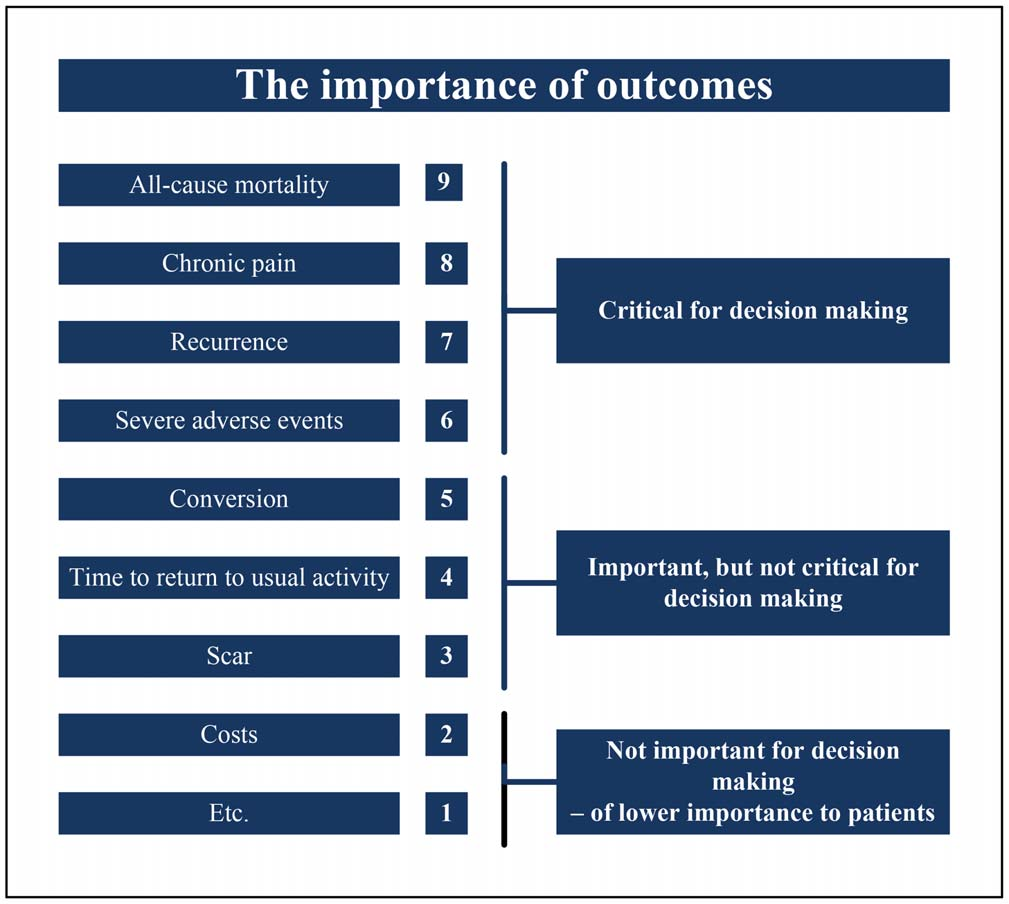
Hierarchy of outcomes according to importance to patients undergoing inguinal hernia repair (GRADE 2004). Some outcome measures may be correlated (e.g. recurrence is included in severe adverse events).

The outcome measures were graded according to the patients' perspective (Figure 3). All-cause mortality, chronic pain, recurrences, and severe adverse events were considered critical for decision making. Other secondary outcomes were graded important, but not critical for decision making.

**Figure 4:**
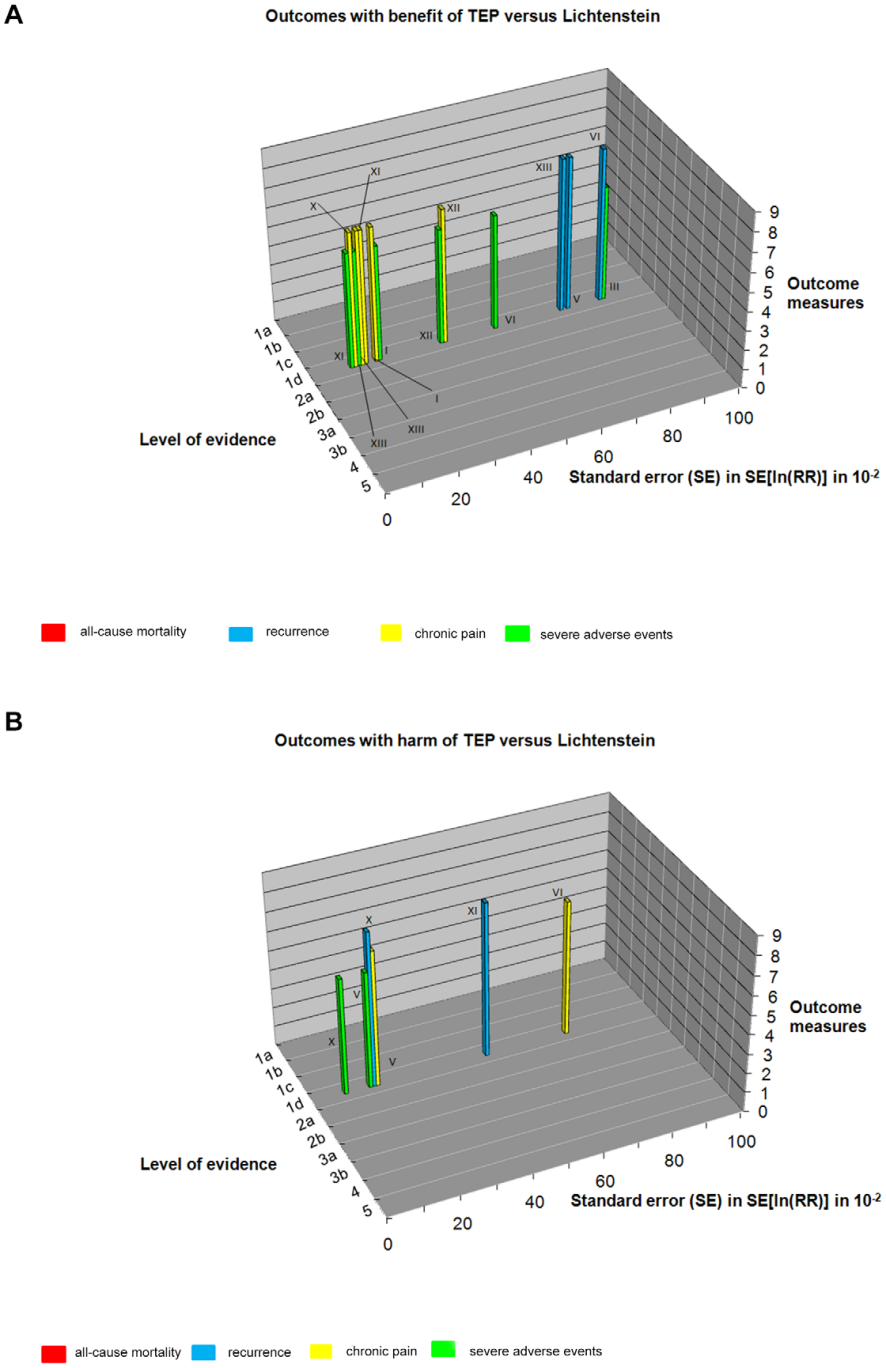
The Manhattan Overview for benefit and harm. 4a: trials and their outcomes with benefit according to the three dimensions; standard error (SE), graded from patients perspective (0–9) and level of evidence (1a–5). See legends for references to trial numbers I–XIII. 4b: trials and their outcomes with harm according to the three dimensions; standard error (SE), graded from patients perspective (0–9) and level of evidence (1a–5). See legends for references to trial numbers I–XIII. Legend for reading Figure 4 The Roman numbers are corresponding to the clinical trials as stated below. I  =  Wright 1996 [41]. II  =  Merello 1997 [25]. III  =  Heikkinen 1998 [26]. IV  =  Moreno 1999 [36]. V  =  Andersson 2003 [28]. VI  =  Colak 2003 [29]. VII  =  Gokalp 2003 [34]. VIII  =  Hildebrandt 2003 [35]. IX  =  Lal 2003 [38]. X  =  Neumayer 2004 [37]. XI  =  Eklund 2006 [30]. XII  =  Lau 2006 [40]. XIII  =  Langeveld 2010 [39].

All trials were assessed as trials with high risk of bias (level of evidence 1d [11]). The standard errors of the meta-analytic estimate were calculated (Table 2). Figure 4a&b shows the three-dimensional ‘Manhattan’ error matrix consisting of the standard error (SE), the level of evidence and the outcome measures.

**Table 2:** ordering of the available evidence.

Trial		Level of evidence	All cause mortality	Standard error					
				Recurrence		Chronic pain		Severe adverse events	
I	Wright 1996 [41]	1d	n/a	n/a	-	0.18	b	0.18	b
II	Merello 1997 [25]	1d	n/a	z	e	n/a	-	z	e
III	Heikkinen 1998 [26], [27]	1d	n/a	z	b	z	b	0.86	b
IV	Moreno 1999 [36]	1d	n/a	z	e	n/a	-	z	e
V	Andersson 2003 [28]	1d	n/a	0.75	b	0.18	h	0.16	h
VI	Colak 2003 [29]	1d	n/a	0.85	b	0.74	h	0.53	b
VII	Gokalp 2003 [34]	1d	n/a	z	e	z	h	z	h
VIII	Hildebrandt 2003 [35]	1d	n/a	z	h	z	h	z	h
IX	Lal 2003 [38]	1d	n/a	z	e	z	h	z	h
X	Neumayer 2004 [37]	1d	z	0.17	h	0.12	b	0.09	h
XI	Eklund 2006 [30]-[33]	1d	n/a	0.50	h	0.15	b	0.12	b
XII	Lau 2006 [40]	1d	n/a	z	e	0.38	b	0.37	b
XIII	Langeveld 2010 [39]	1d	n/a	0.73	b	0.14	b	0.13	b

Ordering of the available evidence according to levels of evidence (systematic error), standard error (random error) and outcome measures (design error) in TEP versus Lichtenstein patients. b = benefit, h = harm, e = equal, z = zero events in one or both intervention arms. n/a =  no data available.

The systematic error distinguishes studies based on their risk of bias. Trials with low risk of bias and data on mortality are absent. At a glance it is noticed that chronic pain was assessed with low risk of random error; in five trials the SE's were between 0.12 and 0.18.

Recurrences are associated with considerable risk of random error (only one trial has SE of 0.17 and all other trials have SE's>0.50). SAE were also assessed with low risk of random error as five trials had SE's between 0.09 and 0.18.

### Effect of interventions

Thirteen trials randomized 5404 patients for inguinal hernia repair between the TEP technique (2684 patients) and Lichtenstein's technique (2720 patients).

### Primary outcomes

#### Mortality

No meta-analysis of all-cause mortality was performed as only one trial reported mortality with merely two deaths in the TEP group [37].

#### Chronic pain

**Figure 5:**
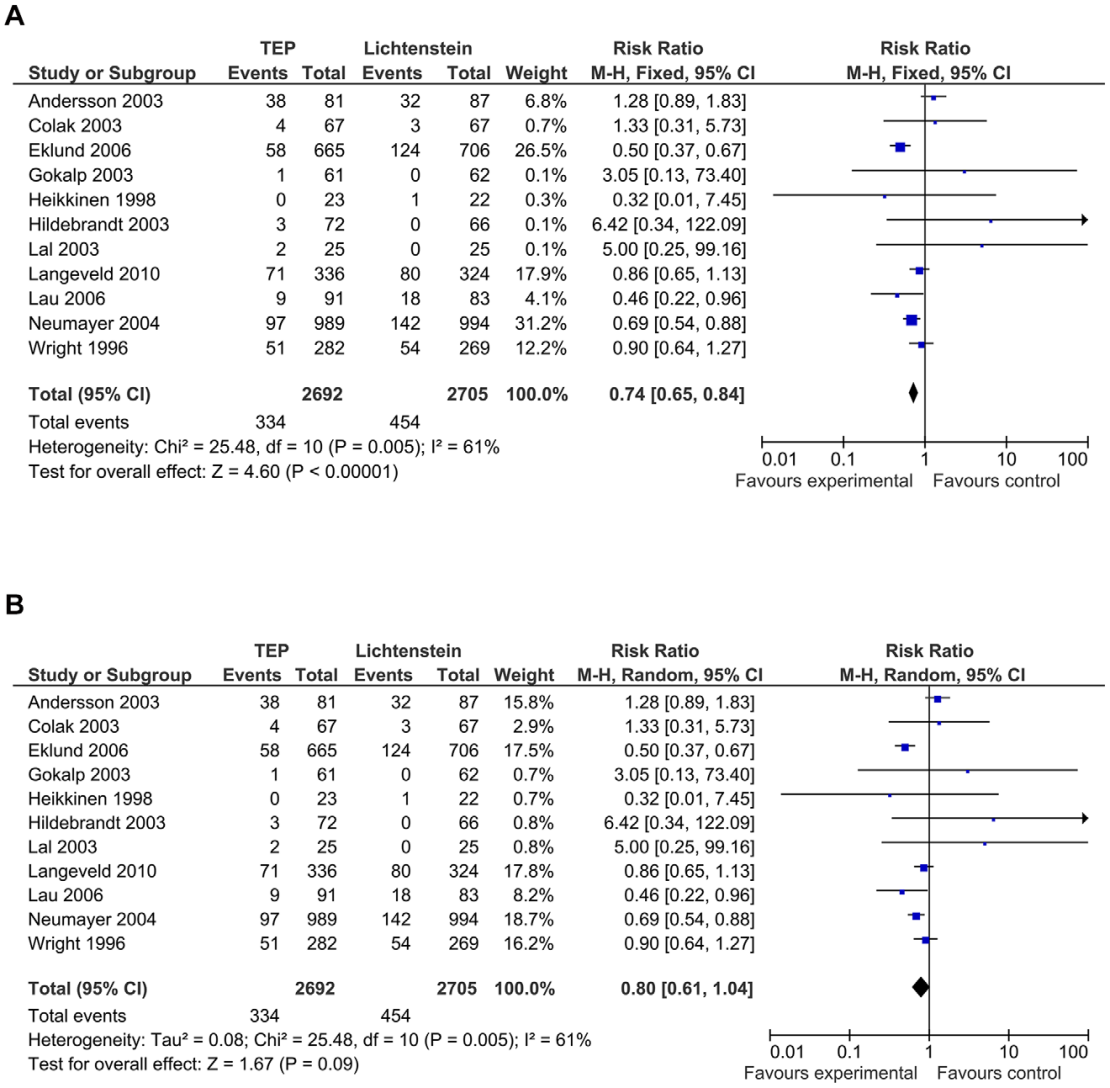
Forest plot of Chronic pain. **5a:** forest plot on chronic pain. Fixed-effect model. **5b:** forest plot on chronic pain. Random-effects model.

Eleven trials reported on chronic pain defined as persisting pain for longer than three months, in 334 patients (12.4%) in 2692 patients in the TEP group versus 454 patients (16.8%) in 2705 patients in the Lichtenstein group. However, substantial heterogeneity was present (I^2^ 61%; *p* = 0.005), and the random-effects model showed no statistically significant differences between the TEP and Lichtenstein group (RR 0.80; CI 0.61 to 1.04; *p* = 0.09). Meta-analysis using the fixed-effect model showed significant less chronic pain using the TEP technique (RR 0.74; CI 0.65 to 0.84; *p*<0.00001) (Figure 5a&b).

**Figure 6:**
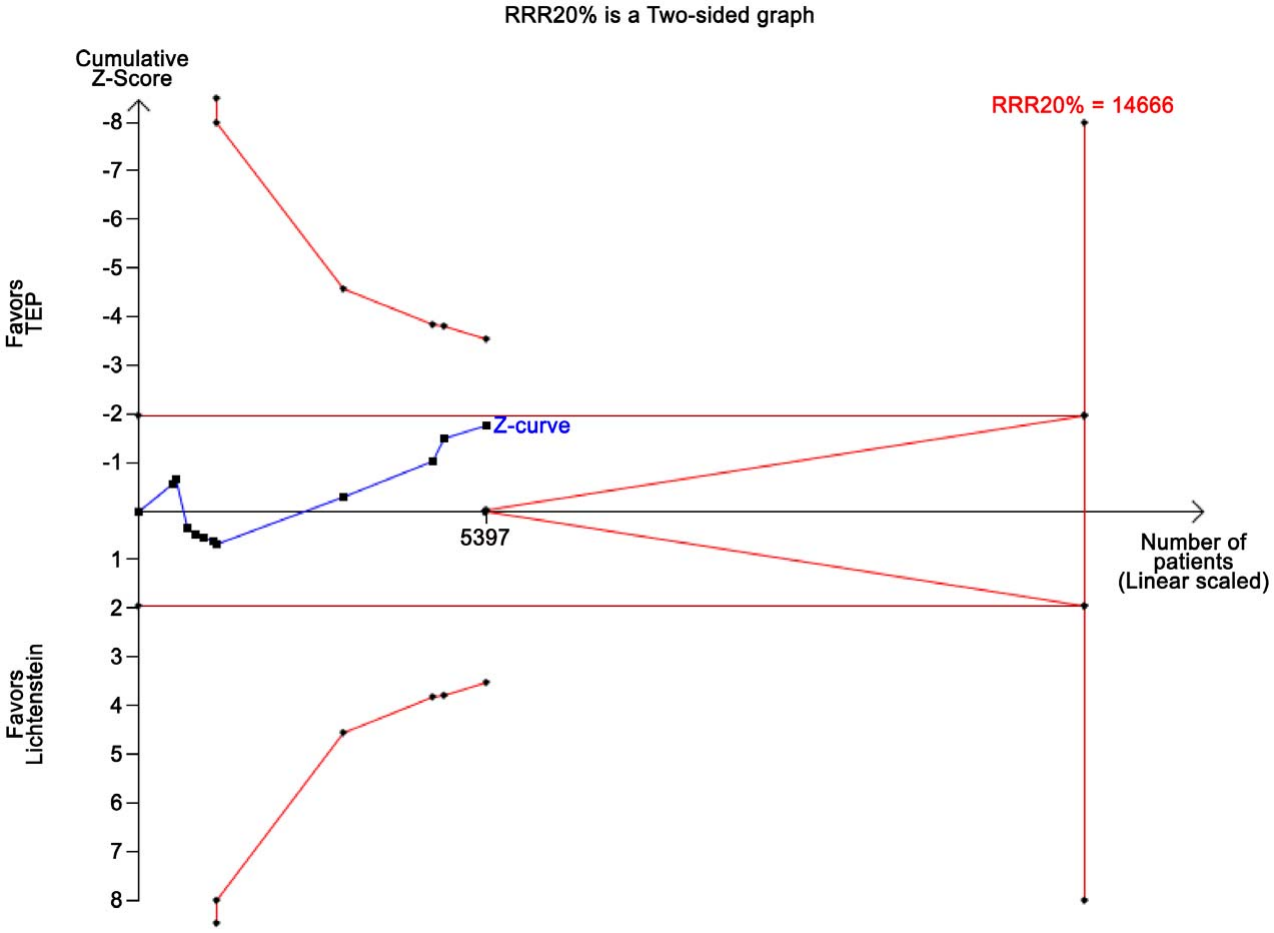
TSA on chronic pain data. Trial sequential analysis of the effect of TEP vs. Lichtenstein anticipating a realistic relative risk decrease of chronic pain of 20% with TEP compared to Lichtenstein assuming a control event proportion of 17% and a type 1 error risk of 5% and a type 2 error risk of 20% (power = 80%). Even in a traditional random-effects meta-analysis the intervention effect is not statistically significant and therefore the cumulative z-curve does not cross the TSMB for harm, constructed for a diversity-adjusted required information size of 14.666 participants either suggesting lack of evidence for TEP reducing the proportion of patients with recurrence.

The TSA, assuming a control event rate of 17%, an anticipated intervention effect of 20% relative risk reduction (RRR), and a power of 80%, shows a cumulative *z*-curve without crossing the TSMB (Figure 6). Moreover, the *z*-curve does not even cross the conventional *p* = 0.05 boundary, showing lack of evidence to conclude on the superiority (or futility) in the comparison of the techniques considering chronic pain.

#### Recurrences

All 13 trials reported on recurrences with 130 recurrences (5.0%) out of 2583 patients in the TEP group versus 72 recurrences (2.7%) out of 2598 patients in the Lichtenstein group.

Meta-analysis using the fixed-effect model showed significant more recurrences in the TEP group (RR 1.89; 95% CI 1.42 to 2.50; *p* = 0.0001).

**Figure 7:**
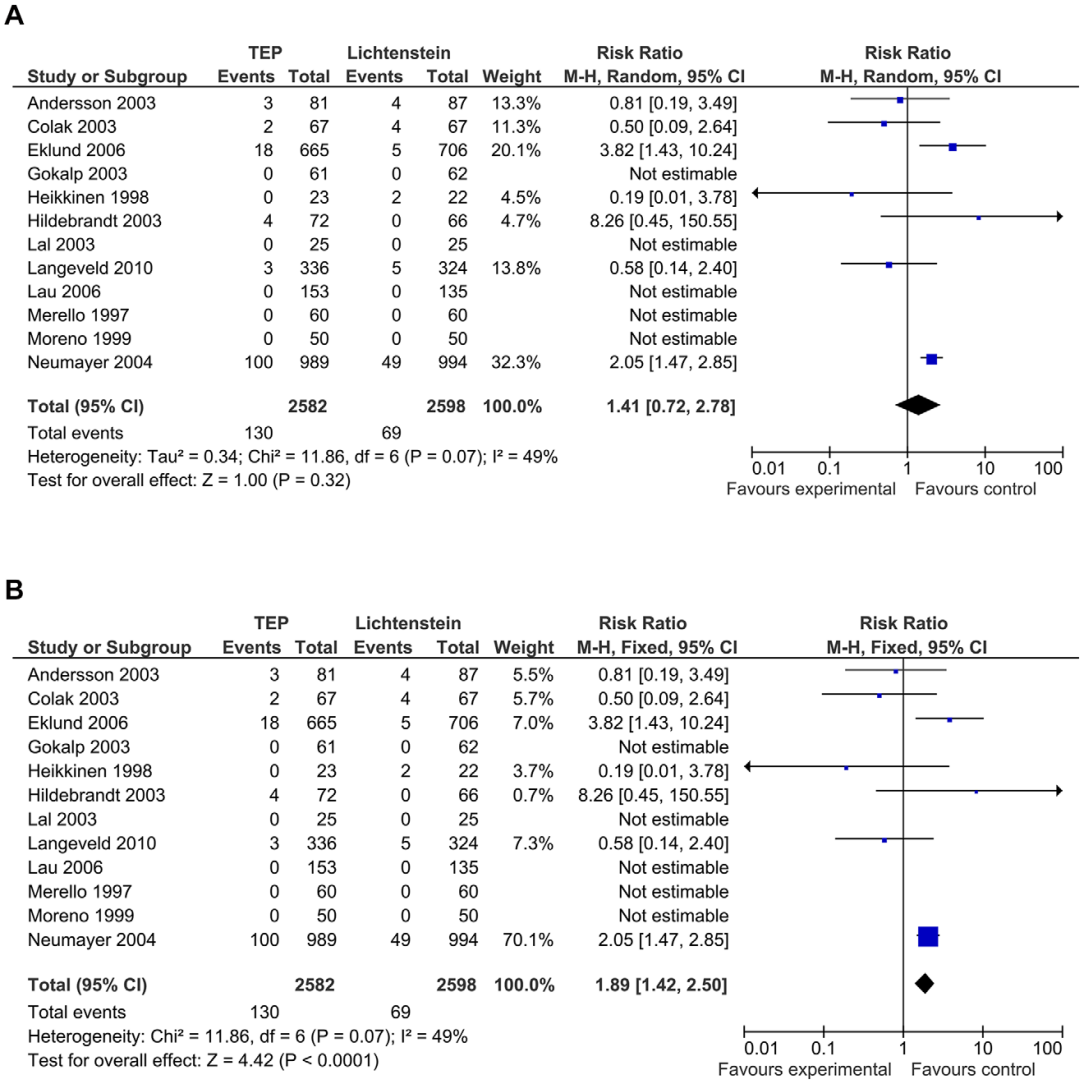
Forest plot on recurrence. **7a:** forest plot on recurrence. Fixed-effect model. **Figure 7b** **:** forest plot on recurrence. Random-effects model.

Random-effects meta-analysis showed no statistically significant difference (RR 1.41; 95% CI 0.72 to 2.78; *p* = 0.32) I^2^ = 49% (Figure 7a,b). Calculations using OR did not show noticeable difference.

**Figure 8:**
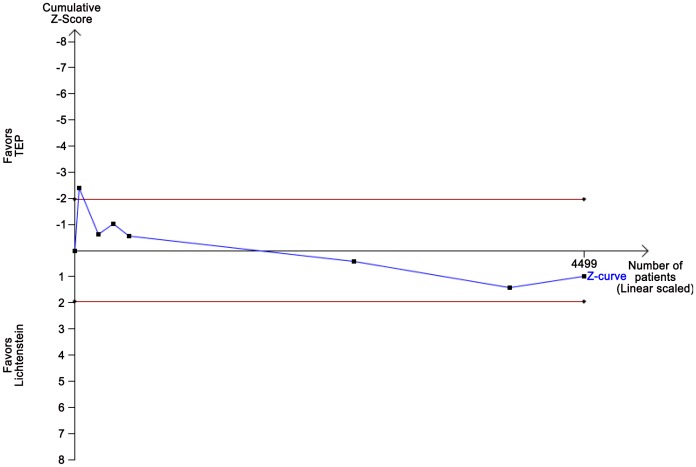
TSA on recurrences TEP versus Lichtenstein. TSA of the effect of TEP vs. Lichtenstein anticipating a realistic relative risk increase of recurrence of 20% with TEP compared to Lichtenstein assuming a control event proportion of 3%, a type 1 error risk of 5%, and a type 2 error risk of 20% (power = 80%). Even in a traditional random-effects meta-analysis the intervention effect is not statistically significant and therefore the cumulative z-curve does not cross the TSMB for harm. The required information size is incalculable due to too little information available, suggesting lack of evidence for TEP reducing the proportion of patients with recurrence.

TSA assuming a control event proportion of 3%, an anticipated intervention effect of 20% RRR, and a power of 80% showed no crossing of either the TSMB, the conventional boundary, or futility boundaries (Figure 8). TSA showed that many more randomized patients are needed before firm evidence can be reached as the diversity adjusted information size is incalculable.

#### Severe adverse events

All 13 trials reported on the composite outcome measure of severe adverse events (SAE) including all serious complications. There were 509 patients (18%) with SAE out of 2811 patients in the TEP group versus 559 patients (20%) with SAE out of 2833 patients in the Lichtenstein group.

Meta-analysis using both the random-effects models (RR 0.91; CI 0.73 to 1.12; *p* = 0.37) (I^2^ = 58%) and the fixed-effect model (RR 0.92; CI 0.83 to 1.02; *p* = 0.12) showed no statistical significant difference between the TEP and the Lichtenstein technique.

**Figure 9:**
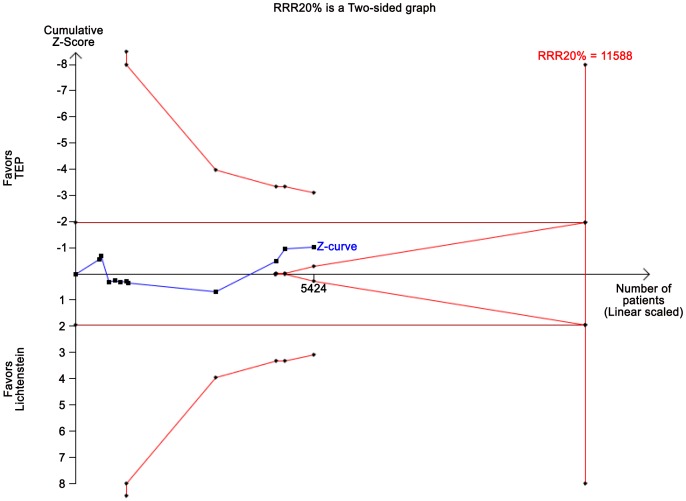
TSA on severe adverse events TEP vs. Lichtenstein. TSA of the effect of TEP vs. Lichtenstein anticipating a realistic relative risk reduction of severe adverse event of 20% with TEP compared to Lichtenstein and assuming a control event proportion of 20% and a type 1 error risk of 5% and a type 2 error risk of 20% (power = 80%). Even in a traditional random-effects meta-analysis the intervention effect is not statistically significant and therefore the cumulative z-curve does not cross the TSMB constructed for a diversity-adjusted required information size of 11.588 participants suggests lack of firm evidence that TEP reduces the proportion of patients with severe adverse events when the analysis adjusts the significance level for considering sparse data and repetitive testing on accumulating data.

TSA assuming a control event proportion of 20%, an anticipated intervention effect of 20% RRR and a power of 80% showed that the cumulative *z*-curve did not cross neither the TSMB the conventional, nor the futility boundaries (Figure 9).

### Secondary outcomes

#### Conversions

Ten of the 13 trials reported conversion. There were 168 patients with conversions (7%) in 2425 patients in the TEP group versus 22 patients with conversions (1%) in 2455 patients in the Lichtenstein group. Meta-analysis using both the fixed- and random effects models showed significantly more conversions in the TEP group (fixed-effect model, RR 6.96; 95% CI 4.58 to 10.58; *p* = 0.00001). No heterogeneity was present (I^2^ = 0%).

**Figure 10:**
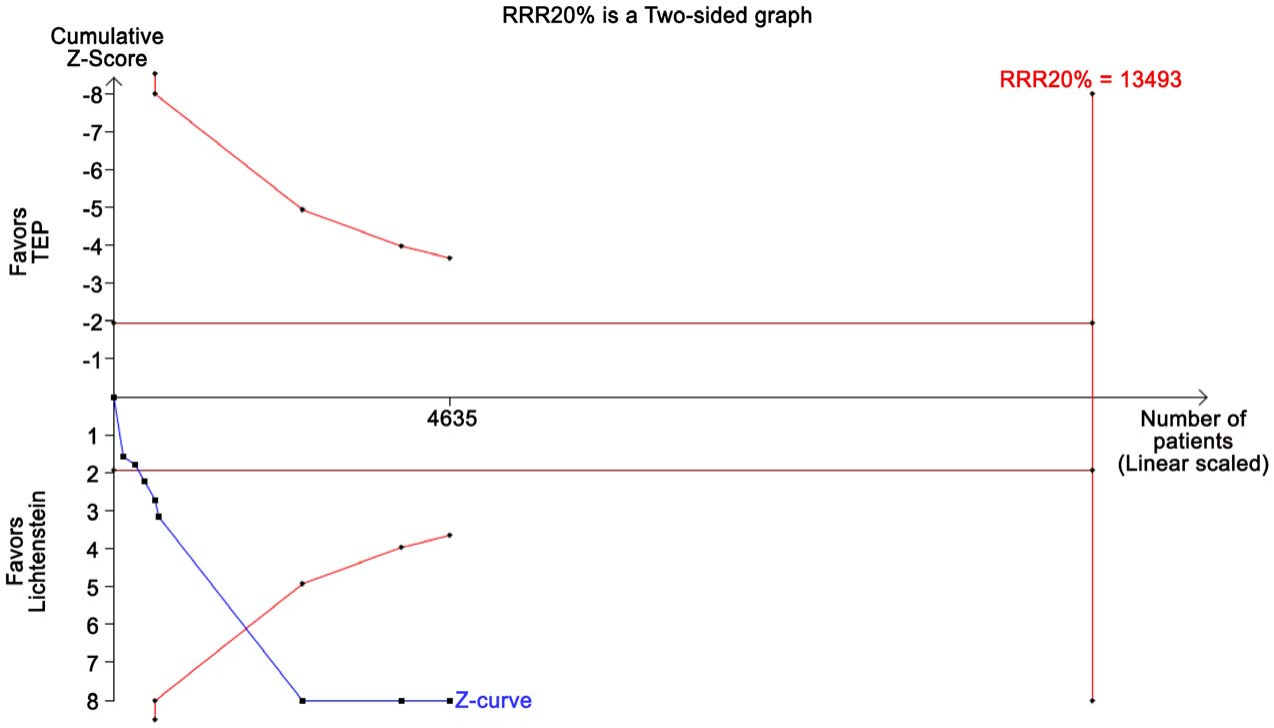
TSA shows more conversions for TEP compared to Lichtenstein.

TSA assuming a control event proportion of 5%, an anticipated intervention effect of 20% RRR and a power of 80% showed that the *z*-curve did cross the TSMB showing firm evidence that TEP is associated with substantially more conversions compared to the Lichtenstein technique (Figure 10).

### Time to return to usual activity, hospital stay and operative time

There was a huge variation in return to usual activity (I^2^ = 78%), hospital stay (I^2^ = 81%), and operative time (I^2^ = 96%) in the included trials. Therefore, pooling of data was not performed.

#### Other outcomes: persisting numbness

Eight trials reported persisting numbness. There were 70 patients (4.3%) with persisting numbness out of 1616 patients in the TEP group versus 205 patients (12.5%) out of 1639 patients in the Lichtenstein group. The random-effects model (I^2^ = 37%) showed significant less persisting numbness when using the TEP technique (RR 0.32; 95% CI 0.21 to 0.49).

TSA assuming a control event proportion of 12%, an anticipated intervention effect of 20% (RRR), and a power of 80% showed that the *z*-curve did cross the TSMB indicating firm evidence, notwithstanding the high bias risk, that TEP is associated with less persisting numbness compared to Lichtenstein.

#### Subgroup analyses

As none of the trials had low risk of bias and trial reports did not clearly mention different anaesthesia techniques, the pre-planned subgroup analyses could not be conducted. No indications were found that the year of publication was associated with any of the outcome results. The funnel plots (Appendix S2) showed no clear arguments for small trial bias including publication bias [chronic pain: Begg's test: *p* = 0.53 (2-tailed); Egger's test: *p* = 0.35 (2-tailed) and SAE: Begg's test: *p* = 0.76 (2-tailed); Egger's test: *p* = 0.60 (2-tailed)].

## Discussion

This systematic review with meta-analysis included thirteen trials randomizing 5404 patients comparing the TEP with the Lichtenstein technique. So far, there is no conclusive evidence of differences in proportions of patients with chronic pain and recurrences between the two techniques. Data have been evaluated according to the three dimensions of risk of error: bias, ‘play of chance’, and design. Trials fall short on the bias protection, the included numbers of patients, and the chosen outcomes. Trial sequential analysis (TSA) and the error matrix approach were used in addition to conventional meta-analytic techniques to reach these conclusions, favoring one technique over the other, based on firm evidence, cannot be drawn yet. There is neither evidence that one technique favors the other nor for a 20% non-inferiority comparing the two techniques.

All trials must be classified as having high risk of bias, as they all scored unclear or high risk of bias in three or more of the eight bias risk components (Figure 2). Therefore, the meta-analytic effect estimates in our analyses may eventually appear to overestimate the effect when trials with low risk of bias emerge 21–23. In this review proportions of SAE are high, 18% and 20%, respectively, in the TEP and Lichtenstein group. These percentages are higher than the maximally reported in other reviews that include non-randomized cohorts [1]. However, this is in concordance with methodological studies showing linkage between unclear/inadequate bias control and risk of significant overestimation of beneficial effects and underestimation of adverse effects [45].

There is substantial risk of random error regarding the primary outcomes of chronic pain, recurrences, and severe adverse events (Table 2 and Figure 4a,b). TSA shows that many more randomized patients may be needed, e.g. 9269 and 6164 respectively, considering chronic pain and SAE before a conclusion on effect or lack of effect can be reached. Recurrence seems to be so rare that the required number of patients needed to identify an effect is incalculable.

In this review the outcome measures were graded from the patients' point of view according to GRADE, focusing on the patient important outcomes critical for decision making [6], [11]. Chronic pain, recurrence and SAE were considered as such critical outcomes [5].

Before the use of a mesh became standard (e.g. Bassini's technique), recurrence was regarded as the most important outcome in inguinal surgery. After non-mesh repair using Bassini's technique at least 8% of patients may experience recurrence [46]. However, after introduction of the mesh the number of patients with recurrence is reported as low as 2% with Lichtenstein's technique [47]. Reduced numbers of patients with recurrence and mesh-associated pain have drawn the attention towards another primary outcome: chronic pain. Up to 40% of patients having chronic pain has been reported recently after the Lichtenstein's technique [48].

It is uncertain whether low-weight or ‘soft’ meshes decrease the number of patients with chronic pain, however, sufficient data on the type of mesh was not available from trials included in this review.

This review focuses on primary outcomes, graded as critical for decision making [6], [11]. Secondary outcomes were not considered to be equally important. Inguinal hernia repair is largely a day-case procedure [49]. Budget restrictions, logistic arguments, surgeon's habits, or organizational procedures may be involved in different cultural situations making comparison and pooling of outcomes like hospital stay and operative time unreliable. Moreover, in the meta-analyses (clinical as well as statistical) heterogeneity appears to be high. Therefore, from the patients' perspective, outcomes like hospital stay and duration of operation should probably be avoided for deciding whether one technique should be preferred for another as long as critical outcomes have not been sufficiently evaluated (Figure 3).

Previous reviews suggest lower proportions of chronic pain associated with TEP [1]. However, these reviews did consider heterogeneous groups of interventions (TEP and TAPP) and they conducted a multitude of post hoc subgroup analyses making conclusions premature and unreliable. Moreover, the superiority of one technique cannot be claimed based on comparisons of heterogeneous groups of interventions. There is still a considerable risk that the advantage of the TEP procedure suggested by the fixed-effect model, ignoring the large heterogeneity, may turn out to be the combined result of bias and random-error.

The included trials did not consider any learning curve effect on both techniques. However, learning curve effects probably do influence effect estimates. The learning curve of the TEP technique may be less steep compared to the Lichtenstein technique, and therefore results of the TEP technique may have been less favorable than expected. It may be that highly experienced and dedicated hernia surgeons in large volume centres produce more favourable results with TEP, regarding the important outcomes from patients' perspective. Residents or non hernia-dedicated surgeons participating in the trials may have produced the heterogeneous results. Therefore, common clinical practice and the number of patients with complication ought to be followed up through clinical databases and compared with benchmark values [3].

After completing this review, it is concluded that chronic pain continues to remain an important issue after hernia surgery. Both techniques (TEP and Lichtenstein) are associated with considerable rates of chronic pain. It has to be established whether the suggested point estimate of the relative risk reduction of approximately 20% of pain and SAE with TEP is actually “free” of bias and random error.

A priori, a composite outcome measure of SAE including chronic pain, deep wound infections, vascular injuries, visceral injuries and recurrences was constructed [5]. This summary outcome may have included patients counted twice since complications are summarized rather than considering the total number of patients with one or more SAE. Although all trial authors were contacted repetitively for additional data, their response rate was low.

However, since the vast majority of patients recover without any SAE it was hypothesized that this sampling error only occurred occasionally.

Future trials and studies should be well argued before they are launched. However, even though databases may provide large numbers of patients, and, given they inform on consecutive cohorts of patients and may provide some answers of the actual status on benefits and harms, they will always be prone to the huge risk of bias introduced by the choice of intervention by indication. None of the trials included in this review are large trials in the sense that they statistically have the power to detect or exclude even rather large intervention effects on important outcomes. Therefore, future studies should plan to check their position along the 3 dimensions of possible errors: bias, ‘the play of chance’ and the choice of outcomes. It has been proven extensively that trials with low risk of bias produce more reliable results compared with trials with high risk of bias [3], [10].

Despite how provocative it may seem and based on the above considerations, it is proposed to conduct a new large trial (or several trials) with low risk of bias and with outcomes critical for decision making. These future trials should focus on comparing techniques each using a preperitoneal mesh position [42], [47], [49], and use the present reference technique as comparator (Table 3 [50]).

**Table 3:** Checklist of recommendations for future randomized clinical trials, comparing the TEP with the Lichtenstein technique.

Item	Recommendation
To avoid bias	The trial report should be able to fulfill the CONSORT statements [50].
To minimize risk of random error	The sample size should be exceed e.g. 2000 patients. It may not be just one trial, but at least the total number of patients accrued in future trials exceed 2000.
To avoid design error	*One* technique, no mixed groups (e.g. just TEP).
Comparator intervention	*One* reference technique (e.g. just Lichtenstein).
Comparison	Outcome measures critical for decision making according to the GRADE [6].
To get the evaluation of serious adverse advents (SAE) right	**Count the patients** with one or more SAE, and not just the total number of SAE. This will lead to less multiple counts and avoid sampling error when the outcome is evaluated.
	This outcome may very well be the most important at the end of the day.
Mesh position	Preperitoneal (sublay) position.

*In an attempt to bridge the information gap a new trial should at least comprise as many patients as the hitherto largest and that preferably several new trials will be needed with at least as many patients as it takes to produce a boundary break through (boundary for benefit, harm or futility) in the TSA, or in the worst case scenario; to close the gap between the required and the presently accrued information size.*

## Supporting Information

Appendix S1 **Presents the search strategy that was followed in the different online libraries, pubmed/medline, the Cochrane library and Embase.** The full key terms and MeSH terms are described.
(PDF)

Appendix S2 **Presents the Funnel Plots on chronic pain and severe adverse events.** The Begg's and Egger's tests are presented (2-tailed). No arguments for small trial bias were found.
(PDF)
