# Correction: The Totally Extraperitoneal Method versus Lichtenstein's Technique for Inguinal Hernia Repair: A Systematic Review with Meta-Analyses and Trial Sequential Analyses of Randomized Clinical Trials

**DOI:** 10.1371/annotation/4775d24d-130e-40f8-a19e-fc4ad5adb738

**Published:** 2013-01-18

**Authors:** G. G. Koning, J. Wetterslev, C. J. H. M. van Laarhoven, F. Keus

There is an error in the legend for Figure 7, "Forest plot on recurrence." The correct Figure 7 legend is: 7a: forest plot on recurrence. Random-effects model. Figure 7b: forest plot on recurrence. Fixed-effect model. 

