# Supplementary material for: The Totally Extraperitoneal Method versus Lichtenstein's Technique for Inguinal Hernia Repair: A Systematic Review with Meta-Analyses and Trial Sequential Analyses of Randomized Clinical Trials
Source: PLoS One. 2013 Jan 11;8(1):e52599. doi: 10.1371/journal.pone.0052599 (PMC3543416; doi:10.1371/journal.pone.0052599)
Supplement: Appendix S1 — Presents the search strategy that was followed in the different online libraries, pubmed/medline, the Cochrane library and Embase. The full key terms and MeSH terms are described. (PDF) [file pone.0052599.s001.pdf]

Appendix S1: search strategy (updated until January 2012).

PubMed/MEDLINE:

("Clinical Trials as topic" [MeSH Terms] OR "Randomized Controlled Trials as Topic" [MeSH Terms] OR random* OR trial) AND (TEP OR TEPP OR (total* AND extraperiton*) OR lichten* OR *liechten* OR laparosc* OR "Laparoscopy" [MeSH Terms] OR preperiton* OR (endosc* AND (inguinal hernia OR "Hernia, Inguinal" [MeSH Terms])))

Full spelling of terms used in the search: randomized controlled trial, totally extraperitoneal, extra peritoneal, Lichtenstein, Liechtenstein, laparoscopy, laparoscopic, preperitoneal, endoscopic, inguinal hernia

CENTRAL (Wiley):

(Inguinal hernia or groin hernia) in Title, Abstract, Keywords, OR Hernia, Inguinal in MeSH descriptor in Trials in the Cochrane Central Register of Controlled Trials (http://www.thecochranelibrary.com).

EMBASE (OvidSP):

1	tep.mp. [mp=title, abstract, subject headings, heading word, drug trade name, original title, device manufacturer, drug manufacturer, device trade name, keyword]<br>with terms: totally extraperitoneal
2	tepp.mp. [mp=title, abstract, subject headings, heading word, drug trade name, original title, device manufacturer, drug manufacturer, device trade name, keyword]<br>with terms: totally extraperitoneal
3	(total* and (extraperiton* or extra periton*)).mp. [mp=title, abstract, subject headings, heading word, drug trade name, original title, device manufacturer, drug manufacturer, device trade name, keyword]<br>with terms: totally extraperitoneal, extra peritoneal
4	(lichtenst* or liechtenst*).mp. [mp=title, abstract, subject headings, heading word, drug trade name, original title, device manufacturer, drug manufacturer, device trade name, keyword]<br>with terms: Lichtenstein, Liechtenstein
5	laparosc*.mp. [mp=title, abstract, subject headings, heading word, drug trade name, original title, device manufacturer, drug manufacturer, device trade name, keyword]<br>with terms: laparoscopy, laparoscopic
6	(pre periton* or preperiton*).mp. [mp=title, abstract, subject headings, heading word, drug trade name, original title, device manufacturer, drug manufacturer, device trade name, keyword]<br>with terms: pre peritoneal, preperitoneal
7	(endosc* and inguinal hernia).mp. [mp=title, abstract, subject headings, heading word, drug trade name, original title, device manufacturer, drug manufacturer, device trade name, keyword]<br>with terms: endoscopic, endoscopy, inguinal, hernia
8	1 or 2 or 3 or 4 or 5 or 6 or 7
9	(random* and trial).mp. [mp=title, abstract, subject headings, heading word, drug trade name, original title, device manufacturer, drug manufacturer, device trade name, keyword]<br>with terms: randomised, randomized, trial, trials
10	8 and 9
