# Supplementary material for: The Totally Extraperitoneal Method versus Lichtenstein's Technique for Inguinal Hernia Repair: A Systematic Review with Meta-Analyses and Trial Sequential Analyses of Randomized Clinical Trials
Source: PLoS One. 2013 Jan 11;8(1):e52599. doi: 10.1371/journal.pone.0052599 (PMC3543416; doi:10.1371/journal.pone.0052599)

Appendix S2: Funnel plots on chronic pain and severe adverse events.

The Funnel plot on chronic pain. (Begg's test: *p*=0.53 (2-tailed); Egger’s test: *p*=0.35 (2-tailed)).

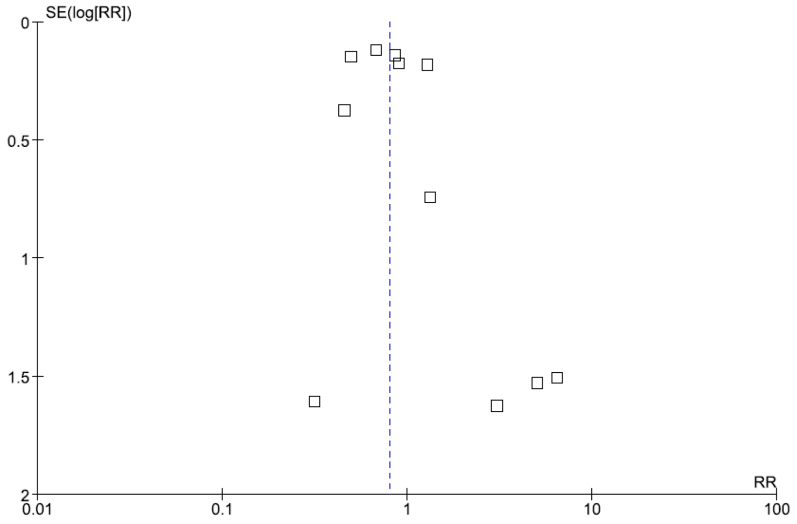

The Funnel plot on severe adverse events (SAE). (Begg's test: *p*=0.76 (2-tailed); Egger’s test: *p*=0.60 (2-tailed)).

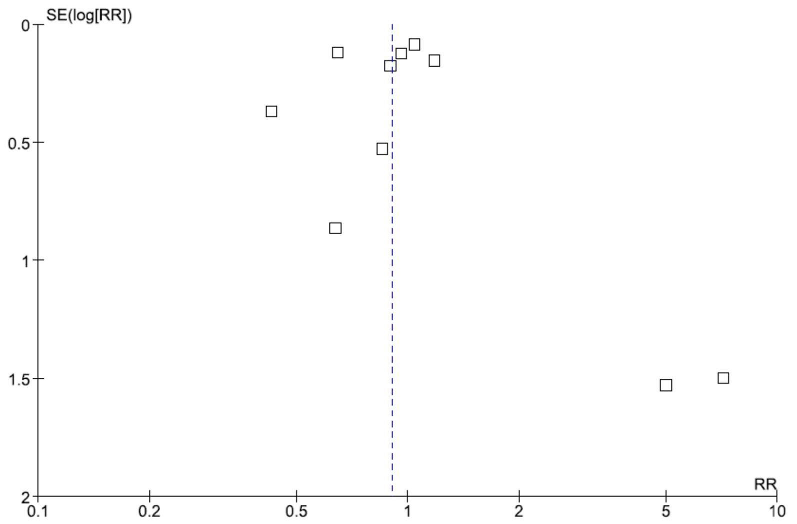

Supplement: Appendix S2 — Presents the Funnel Plots on chronic pain and severe adverse events. The Begg's and Egger's tests are presented (2-tailed). No arguments for small trial bias were found. (PDF) [file pone.0052599.s002.pdf]
